# Solvent Accessibility of Residues Undergoing Pathogenic Variations in Humans: From Protein Structures to Protein Sequences

**DOI:** 10.3389/fmolb.2020.626363

**Published:** 2021-01-07

**Authors:** Castrense Savojardo, Matteo Manfredi, Pier Luigi Martelli, Rita Casadio

**Affiliations:** ^1^Biocomputing Group, Department of Pharmacy and Biotechnologies, University of Bologna, Bologna, Italy; ^2^Institute of Biomembranes, Bioenergetics and Molecular Biotechnologies of the National Research Council, Bari, Italy

**Keywords:** solvent accessible surface area, relative solvent accessibility, protein variations, prediction of solvent accessible surface, pathogenic protein variations

## Abstract

Solvent accessibility (SASA) is a key feature of proteins for determining their folding and stability. SASA is computed from protein structures with different algorithms, and from protein sequences with machine-learning based approaches trained on solved structures. Here we ask the question as to which extent solvent exposure of residues can be associated to the pathogenicity of the variation. By this, SASA of the wild-type residue acquires a role in the context of functional annotation of protein single-residue variations (SRVs). By mapping variations on a curated database of human protein structures, we found that residues targeted by disease related SRVs are less accessible to solvent than residues involved in polymorphisms. The disease association is not evenly distributed among the different residue types: SRVs targeting glycine, tryptophan, tyrosine, and cysteine are more frequently disease associated than others. For all residues, the proportion of disease related SRVs largely increases when the wild-type residue is buried and decreases when it is exposed. The extent of the increase depends on the residue type. With the aid of an in house developed predictor, based on a deep learning procedure and performing at the state-of-the-art, we are able to confirm the above tendency by analyzing a large data set of residues subjected to variations and occurring in some 12,494 human protein sequences still lacking three-dimensional structure (derived from HUMSAVAR). Our data support the notion that surface accessible area is a distinguished property of residues that undergo variation and that pathogenicity is more frequently associated to the buried property than to the exposed one.

## Introduction

In structural bioinformatics, Solvent Accessible Surface Area (SASA) [or briefly Accessible Surface Area (ASA)] of proteins has always been considered a main feature for determining protein folding and stability. Early computational studies (Lee and Richards, 1971; Chothia, 1976; Miller et al., 1987, and references therein) emphasized the role of solvent exposed vs. non-exposed amino acid residues in determining the protein structure. Typically, ASA is defined as the polar solvent accessible area of a given protein, and it is computed by means of a solvent molecule, which probes the protein surface beyond the van der Waals radius. After the first rolling ball algorithm (Shrake and Rupley, 1973), many alternatives became available for computing ASA from the atomic coordinates of the protein in its folded and unfolded state [for review see Ali et al. (2014)]. Evidently, ASA is a function of the three dimensional structure of the protein and, based on ASA values, amino acid residues of a protein can be classified as buried or exposed (Kabsch and Sander, 1983), a property that is conserved through evolution in protein families (Rost and Sander, 1994). ASA is routinely computed as an absolute value or as Relative Solvent Accessibility (RSA), when the ASA value is divided by the maximum possible solvent accessible surface area of the residue (Tien et al., 2013). ASA gained also a pivot role in detecting protein-protein interfaces of molecular complexes in the Protein Data Bank (PDB) [for review see Savojardo et al. (2020), and references therein].

With the advent of machine and deep learning-based approaches (Baldi, 2018), many methods became available for predicting RSA and ASA. They differ mainly in the machine learning approach, the volume of the database of protein structures and the predicted output (ASA, RSA, or binary classification) (Rost and Sander, 1994; Pollastri et al., 2002; Drozdetskiy et al., 2015; Ma and Wang, 2015; Fan et al., 2016; Wu et al., 2017; Kaleel et al., 2019; Klausen et al., 2019).

Surface accessible area of residues can be important also for functional annotation of disease related protein variants. However, this property has been rarely included into the physico-chemical characteristics adopted to describe the residues undergoing variations (Chen and Zhou, 2005; Martelli et al., 2016; Savojardo et al., 2019).

In this study, we investigate the relation between the pathogenicity of human protein variations and the solvent exposure of the residues undergoing variation (wild-type residues). To this aim, we provide an updated version of a highly curated dataset of Single Residue Variations (SRVs) occurring in human proteins that can be mapped in high-quality structures deposited in the Protein Data Bank (PDB). The dataset, here referred to as HVAR3D-2.0, is generated from data available at the HUMASVAR database and builds on top of data previously analyzed in a different study (Savojardo et al., 2019). On this structural dataset, we explore the relationship between pathogenicity of SRVs and the solvent accessibility of the corresponding wild-type residues. In particular, we determine that the majority (67%) of disease-related SRVs occur in buried positions whereas neutral SRVs occur mostly (64.3%) in exposed residues. Moreover, SRVs targeting specific residue types such as glycine, tryptophan, tyrosine, and cysteine, are more frequently associated with disease than others are. Finally, for all residues, and in particular for asparagine, glutamine, histidine, and lysine, the proportion of disease related SRVs largely increases when the wild-type residue is buried, and decreases when it is exposed, confirming that, among other factors, the context can be associated to the pathogenicity of the variations (Casadio et al., 2011).

We extended the above analysis to a larger set of variations included in HUMSAVAR and collected in a dataset called HVARSEQ. In order to estimate the solvent accessibility of all residues undergoing disease-related or neutral SRVs in human proteins, we developed an in-house method based on deep-learning for predicting solvent exposure from sequence. Our method performance is comparable to state-of-the-art methods. We apply it to all the residues of human protein sequences, undergoing pathogenic and neutral SRVs in HVARSEQ.

Results of the large-scale analysis on protein sequences support what observed in protein structures and confirm the different distribution buried/exposed wild-type residues in disease-related and neutral SRVs. Our data suggest that solvent accessibility is a distinguished property of wild type residues undergoing pathogenic variations.

## Materials and Methods

### Variation Databases

All human Single-Residue Variations (SRVs) were collected from HUMASVAR version 2020_04 (Aug 2020). As a first filtering step, we retained variations labeled as “Disease” and “Polymorphism,” neglecting all variations labeled as “Unclassified.” Disease-related SRVs not associated with OMIM diseases were excluded. After this procedure we ended up with a large set of SRVs occurring on human protein sequence. Here this dataset is referred to as HVARSEQ (Human VARiations in SEQuences)

In order to build the structural dataset (here referred to as HVAR3D-2.0, Human VARiations in three Dimensional structures), we firstly identified, among all the sequences included in HVARSEQ, the subset of proteins endowed with a PDB structure meeting the following criteria:

- Coverage of the corresponding UniProtKB sequence is ≥70%;
- Experimental method is X-ray crystallography;
- Resolution is ≤ 3Å.

The mapping of SRV positions on protein structure was performed using data from the Structure Integration with Function, Taxonomy and Sequence (SIFTS) project^1^. Protein structures having ambiguous or wrong SIFTS mapping files were excluded from the dataset.

### Computing Solvent Exposure

The absolute Accessible Surface Area (ASA) of each wild-type residue undergoing variation has been computed using the DSSP program (Kabsch and Sander, 1983). Relative Solvent Accessibility (RSA) values were then obtained dividing absolute ASA values in Å^2^ by residue-specific maximal accessibility values, as extracted from the Sander and Rost scale (Rost and Sander, 1994). Finally, each residue has been classified as buried (B) if its RSA was below 20%, and exposed (E) otherwise.

### Computing P_D_, P_D|R_, P_D|B,R_, and P_D|E,R_

In this study, the background probability of a wild-type residue to be disease associated in a dataset of wild-type residues is computed as follows:

(1) $$\begin{array}{r} P_{D}=\frac{n_{D}}{N} \end{array}$$

where *n*_*D*_ and *N* are the number of wild-type residues undergoing disease-related variations and the total number of wild-type residues undergoing variations (disease related or not) in the dataset, respectively.

The conditional probability of being disease related when variated, given a wild-type residue R, is computed as follows:

(2) $$\begin{array}{r} P_{D|R}=\frac{n_{DR}}{n_{R}} \end{array}$$

where *n*_*DR*_ and *n*_*R*_ are the number of wild-type residues of a given R type, which are disease related upon variations, and the total number of R residues in the whole dataset, respectively.

The conditional probability of a wild-type residue R to be disease related upon variation when buried is computed as:

(3) $$\begin{array}{r} P_{D|B,R}=\frac{n_{DBR}}{n_{BR}} \end{array}$$

where *n*_*DBR*_ and *n*_*BR*_ are the number of buried wild type R residue in the set of wild type disease related upon variation and the total number of buried R wild type residues, respectively.

Similarly, the conditional probability of a wild-type residue R to be disease related upon variation when exposed is computed as:

(4) $$\begin{array}{r} P_{D|E,R}=\frac{n_{DER}}{n_{ER}} \end{array}$$

where *n*_*DER*_ and *n*_*ER*_ are the number of exposed wild type R residue in the set of wild-type disease related upon variation and the total number of exposed R wild type residues, respectively.

All the above probabilities are estimated considering the structural dataset HVAR3D-2.0, and by computing the residue solvent accessibility with the DSSP program. Moreover, we extended the analysis to the whole HVARSEQ sequence dataset, by estimating the residue exposure state (buried and exposed) with a predictor implemented in-house and described in the following section.

### Predicting Solvent Accessibility From the Protein Sequence

The method implements a deep-learning architecture processing an input based on the following descriptors:

- The residue one-hot encoding, representing primary sequence information;
- Evolutionary information encoded with a protein sequence profile, as extracted from multiple sequence alignment generated using the HHblits version 3 program (Steinegger et al., 2019). We performed two search iterations with default parameters against the Uniclust30 database (Mirdita et al., 2017).

Our deep architecture processes the input using three cascading Bidirectional Long-Short Term Memory (BLSTM) layers (Graves and Schmidhuber, 2005). BLSTMs belong to the class of LSTM (Hochreiter and Schmidhuber, 1997), a special recurrent neural network architecture well-suited for processing protein sequence data and extracting significant sequential relations between elements of the sequence. BLSTMs are an extension of LSTMs performing a double scanning of the input sequence, from left to right and vice versa, in order to better capture the sequential relations among sequence positions. The adoption of the recurrent BLSTM allows the method to take into consideration the local sequence context without the explicit use of a fixed-size window centered on each residue.

The output of the third recurrent layer is then provided as input to a time-distributed fully connected layer adopting a sigmoid activation function. This layer is responsible for the final, binary classification of each residue in the sequence into buried or exposed classes. In particular, the numerical output value in the range [0, 1] attached to each residue is interpreted as a probability *p* of being exposed: all residues with *p* ≥ 0.5 are predicted as exposed while those with *p* < 0.5 are classified as buried.

The dataset adopted to train and test the predictor presented in this study has been extracted from the Protein Data Bank (interrogated Oct 15, 2019) (Berman, 2000). Overall, the dataset comprises 2532 non-redundant, author-declared functional monomeric PDB structures, obtained with X-ray crystallography at < 2.5 Å resolution and covering more than 70% of corresponding UniProtKB sequences. All proteins in the dataset share <30% sequence identity. This dataset was then randomly split into a training set, comprising 2,352 sequences, and an independent blind test set including 200 sequences. Proteins in the training set were further split into 10 equally-sized sets for setting the values of hyperparameters with a cross-validation procedure.

Solvent exposure for training/testing data has been computed using DSSP as detailed in Section: Computing solvent exposure. The residues were classified into buried and exposed using a RSA threshold of 20%. Using this threshold, the set of residues is roughly divided into equally sized subsets comprising 52% and 48% of buried and exposed residues, respectively, providing balanced datasets for training and testing.

## Results

### HVAR3D-2.0: A Dataset of Variations Covered by 3D Structure

The structural dataset collected in this work, here referred to as HVAR3D-2.0, is an updated version of the dataset described in a previous study (Savojardo et al., 2019). The dataset has been derived by mapping on PDB structures OMIM-related and neutral SRVs annotated in the HUMSAVAR database^2^, release 2020_08 (Aug, 2020). Only structures determined with X-ray crystallography with resolution ≤3 and covering ≥70% of the corresponding UniProtKB sequences were selected. After this stringent filtering, we ended-up with a high-quality dataset comprising 10,760 human SRVs occurring on 1,255 PDB entries (corresponding to 1,285 protein chains). The set includes 6,778 and 3,982 disease-related and neutral SRVs, respectively. Table 1 lists a summary of the HVAR3D-2.0 content. The HVAR3D-2.0 dataset is available in Supplementary Table 1 in TSV format.

**Table 1:** Statistics of HVAR3D 2.0 dataset.

**Description**	**Counts (#)**
PDB structures	1,255
PDB chains	1,285
Distinct SRV positions	9,379
SRVs	10,760
Disease-related SRVs	6,778
Neutral SRVs	3,982

In the present study, we are interested in investigating the relation between the pathogenicity of SRVs and the solvent accessibility of the residue undergoing variation. For this reason, we firstly computed Accessible Surface Area (ASA) values for all 1,285 protein chains included in the HVAR3D dataset using the DSSP program (Kabsch and Sander, 1983). Raw ASAs were then converted into Relative Solvent Accessibility (RSA) values using the Rost and Sander maximal accessibility scale (Rost and Sander, 1994). Finally, all residues with RSA ≥ 20% were labeled as exposed (E) or buried (B) otherwise. This threshold (or similar ones, in the range of 15–25% RSA) is routinely adopted for computing the protein surfaces and deriving classification datasets in many studies (Thompson and Goldstein, 1996; Mucchielli-Giorgi et al., 1999; Pollastri et al., 2002; Kaleel et al., 2019), since it roughly divides the set of residues in a protein in two equally-sized subsets. In HVAR3D, using a 20% RSA threshold, we obtain 55% and 45% of residues classified as buried and exposed, respectively, corresponding to a realistic characterization of the protein interior (accounting for completely and partially buried residues) and surface (Miller et al., 1987). Preliminary analysis highlighted that the choice of the RSA threshold (in the reasonable range of 15–25% RSA) only minorly affects the conclusions drawn in this study (data not shown). For this reason, all the subsequent analyses were performed using the aforementioned threshold.

**Figure 1:**
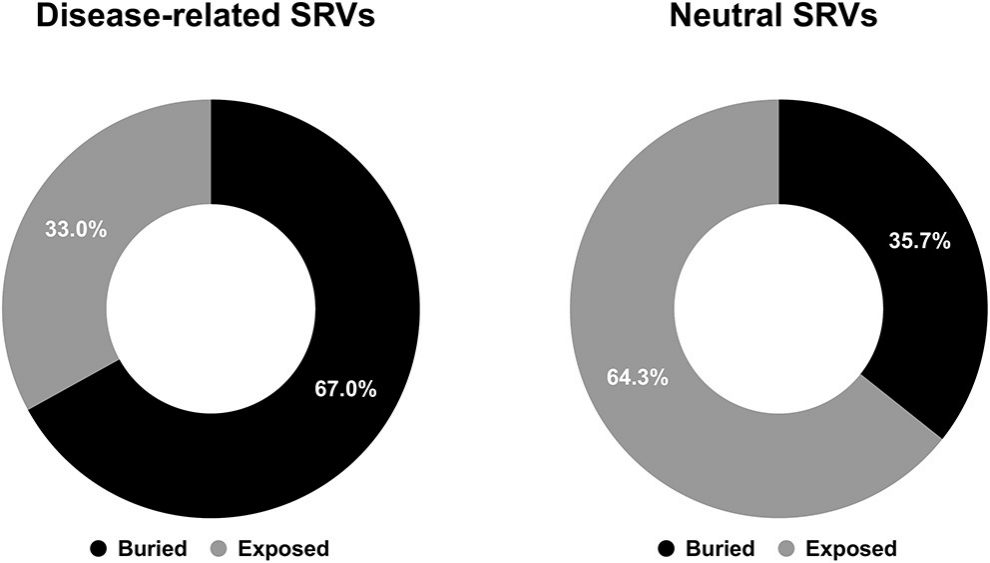
Pie charts showing the fractions of buried/exposed wild-type residues undergoing disease-related (left) and neutral (right) SRVs in the HVAR3D-2.0 dataset, respectively.

Focusing our attention to structure positions undergoing SRVs, we firstly computed the different proportions of buried and exposed wild-type residues associated to disease-related and neutral SRVs. As shown in Figure 1, 67% of wild-type residues undergoing disease-related variations are located in buried positions and about 64% of wild-type residues involved in neutral variations are exposed. This conclusion corroborates, on a much larger structural database, results partially reported in previous studies (Martelli et al., 2016; Savojardo et al., 2019). The relative abundance of disease-related variations in buried positions of the protein and of neutral ones in exposed positions suggests that the solvent accessibility of the variated position is a further property to consider when determining the pathogenicity of a variation.

### Analyzing Distributions of Variated Wild-Type Residues in the Structure Database

**Figure 2:**
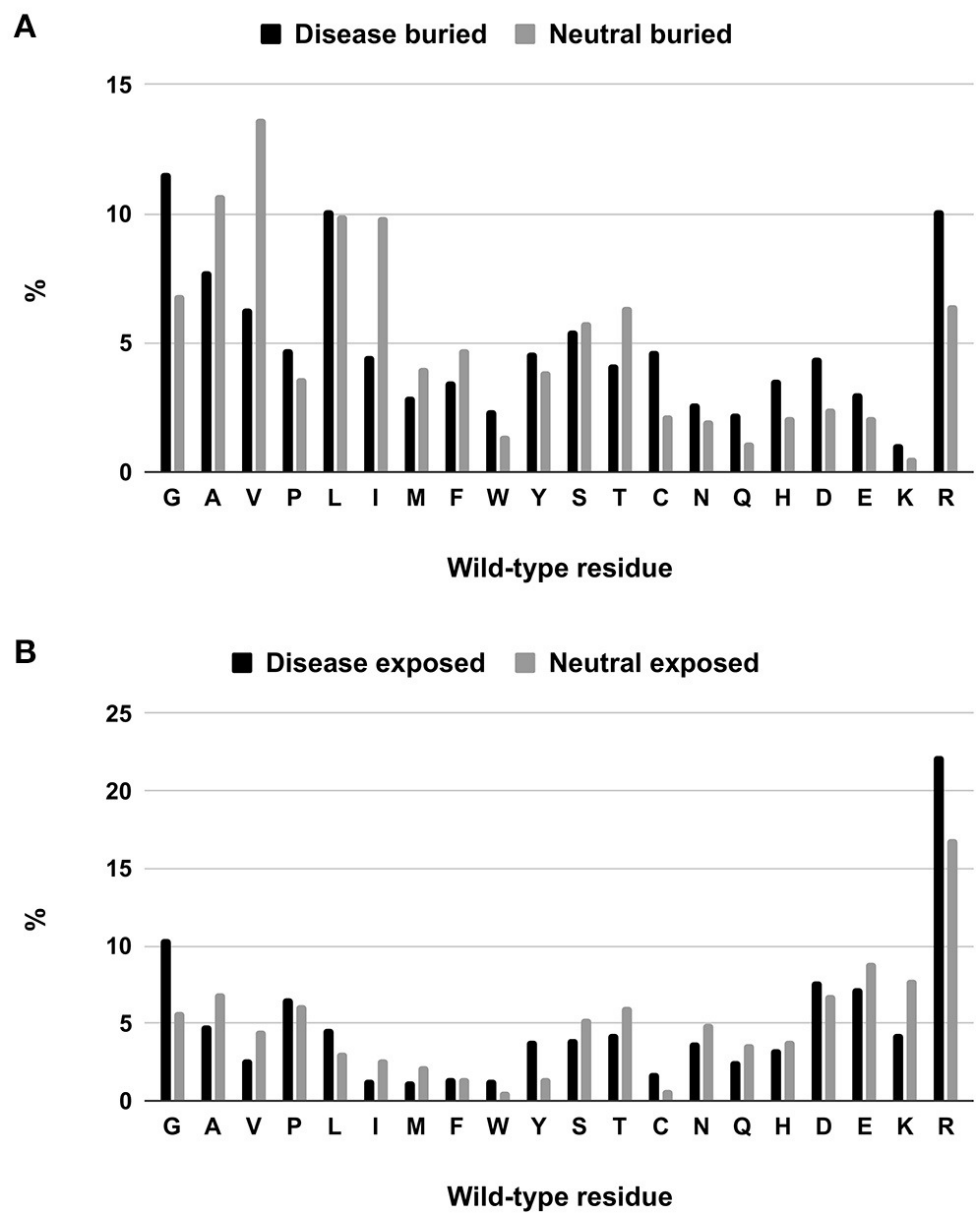
Composition of buried **(A)** and exposed **(B)** wild-type residues undergoing disease-related and neutral variations in the HVAR3D-2.0 dataset.

We tackle the problem of associating solvent exposure to a specific wild-type residue as a characteristic feature to be associated to its variation type (neutral or disease related). We compute the relative frequency of occurrence in the buried and exposed sets of each residue undergoing a disease related or neutral variation (Figures 2A,B). It is evident that while some residue types are more often disease related when variated in the buried state (Q, H, D, E, K), others (including G, W, C, and R) are disease related upon variation in either state.

**Figure 3:**
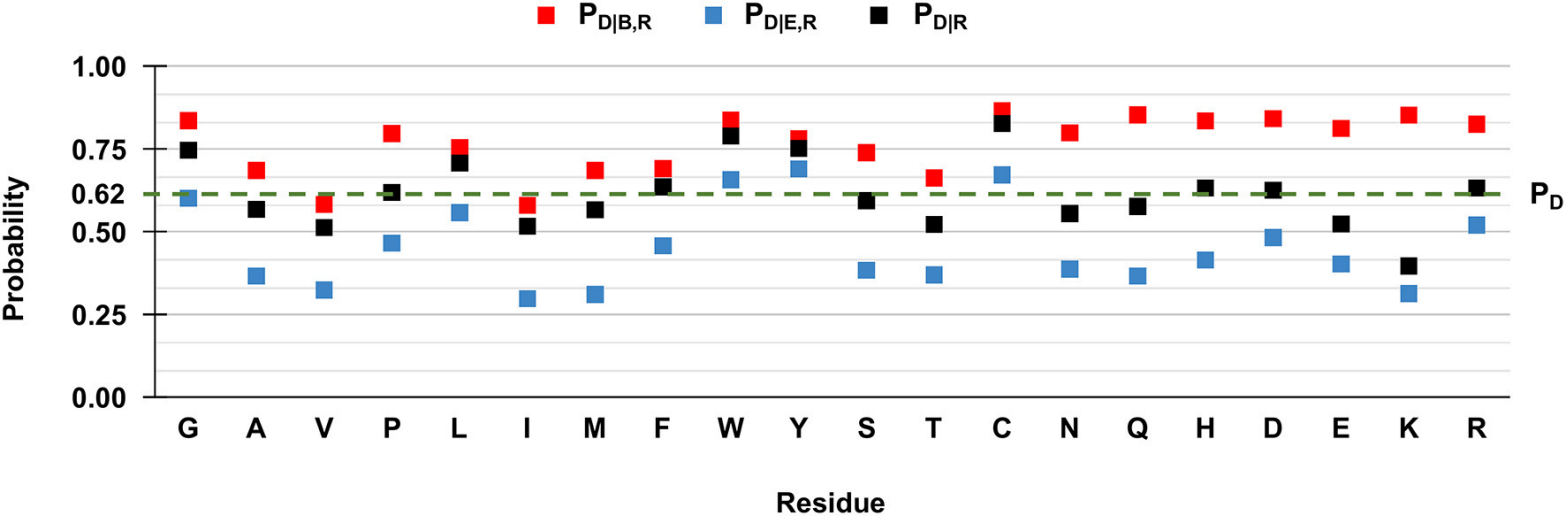
Probabilities of the 20 wild-type residues undergoing disease-related variations, depending on the wild type residue and the exposure state in HVAR3D-2.0. Buried and exposure state of each residue position are estimated with DSSP as described in Section: Analyzing distributions of variated wild-type residues in the structure database. **P**_**D**_: the probability of a wild-type residue (position) to be disease associated in the HVAR3D-2.0 dataset [see Equation (1)]. **P**_**D|R**_: the conditional probability of being disease related when variated, given a wild-type residue [see Equation (2)]. **P**_**D|B,R**_: the conditional probability of a wild-type residue to be disease related upon variation when buried [see Equation (3)]. **P**_**D|E,R**_: the conditional probability of a wild-type residue to be disease related upon variation when exposed [see Equation (4)].

However, when we compute the conditional probabilities per residue type, clearly the tendency of the majority of the wild-type residues is that of being disease-related upon variation when buried (red squares in Figure 3). Indeed, in Figure 3 we show to which extent the knowledge of the solvent exposure changes the *a priori* probability of a given residue type to be associated with disease. For each residue type R, we report the conditional probability of being associated to disease (P_D|R_, black squares) and how the two conditional probabilities (P_D|B,R_ and P_D|E,R_ in red and blue squares, respectively) change, given that the variated residue is buried or exposed. We contrast these values to the baseline frequency of disease related variations in the HVAR3D-2.0 dataset, referred to as P_D_ and equal to 0.62.

In Figure 3, when comparing P_D|R_ of each residue R (black squares) with the baseline value P_D_, it is evident that not all the residues are equally likely to be associated with disease when variated. Residues like glycine (G), leucine (L) tryptophan (W), tyrosine (Y), and cysteine (C) show values of P_D|R_ that are higher than the baseline, indicating that their variations are frequently associated to disease in the database. Furthermore, for all residues the relation P_D|B,R_ > P_D|R_ > P_D|E,R_ holds. This means that for all residue types, the probability that SRVs are related to disease is higher when the wild-type residue is buried (red squares) than when it is exposed (blue squares). The extent of this difference depends on the residue type and it is remarkable for asparagine (N), glutamine (Q), histidine (H), and lysine (K). All these residues are polar and abundant on the protein surface (data not shown). On average, when variated, they are associated to disease with a frequency comparable or lower than the baseline 0.62. However, when variations of these residue types occur in buried positions, the frequency of disease related variations raises to values around 0.8, reaching 0.85 in the case of glutamine (Q) and lysine (K). Remarkably, for three residues [tryptophan (W), tyrosine (Y) and cysteine (C)] the frequency of disease-related variation is higher than the baseline, rather independently of the exposure state. Conversely, the fraction of disease-related variations of valine (V) and isoleucine (I) is lower than the baseline, independently of their accessibility.

Overall, these findings highlight a relation between the pathogenicity of the variation and the solvent accessibility of the wild-type residue and show that the extent of the association depends on the residue type. In all cases, variations occurring in buried positions are more likely to be disease-related. This is particularly so for charged residues, for polar residues such as asparagine (N), glutamine (Q) and histidine (H), and for proline (P), cysteine (C), and tryptophan (W).

### HVARSEQ: A Dataset of Protein Sequences With Variations

Here we make use of computational prediction of solvent accessibility to extend our analysis to all the positions undergoing variations contained in HUMSAVAR. From the HUMSAVAR database, release 2020_08 (Aug, 2020), we collected all polymorphisms and all OMIM-related SRVs occurring in protein sequences. Unclassified SRVs were filtered-out from the set. Overall, 69,385 SRVs were collected. 29,949 and 39,436 SRVs are disease-related and neutral, respectively, occurring on 12,494 protein sequences. Here, this extended set of protein sequences is referred to as HVARSEQ. In Table 2 we summarize the basic statistics of the dataset. The HVARSEQ dataset is available in Supplementary Table 2 in TSV format.

**Table 2:** Statistics of HVARSEQ dataset.

**Description**	**Counts (#)**
UniProtKB sequences	12,494
Distinct SRV positions	64,869
SRVs	69,385
Disease-related SRVs	29,949
Neutral SRVs	39,436

### Predicting Solvent Accessibility

For computing solvent accessibility from protein sequences, we implemented an in-house method for predicting solvent exposure from sequence. The method is based on deep-learning processing of several input features, which encode the protein sequence and the sequence profile (see Materials and Methods for more details on the method). Our method classifies each residue of the sequence into two classes: buried (B), corresponding to residues whose RSA is lower than 20%, and exposed (E), corresponding to residues with RSA ≥ 20%.

Performances are listed in Table 3 and are evaluated adopting three different testing sets (by adopting a cross validation procedure (leftmost column); on the blind test (central column); on our HVAR3D-2.0 dataset, for which solvent exposure can be directly computed using DSSP). Comparing the first two columns, it is evident that our method is robust, achieving generalization performances that are as good and even better than cross-validation results. Overall, our method is able to discriminate buried from exposed residues with Q_2_ (accuracy), MCC (Matthew Correlation Coefficient) and F1 equal to 82%, 0.63 and 82%, respectively. When scored on the HVAR3D-2.0 dataset, the performance is almost unchanged, suggesting that our method is quite stable across different datasets.

**Table 3:** Performance of our deep learning-based method for predicting solvent exposure from protein sequence.

**Scoring index**	**Dataset**		
	**Cross-validation**	**Blind test**	**HVAR3D-2.0**
MCC	0.63	0.63	0.60
Q2 (accuracy)	81%	82%	80%
F1	81%	82%	80%

We also performed a side-by-side comparison between our method and two state-of-the-art approaches, namely PaleAle5.0 (Kaleel et al., 2019) and NetSurfP-2.0 (Klausen et al., 2019). Results are reported in Table 4. All methods perform quite well, with comparable scoring indexes. It is worth mentioning that the testing set used in this benchmark is non-redundant only with respect to our training set: this condition is not guaranteed for the other two methods evaluated, which adopt different training sets. In general, we can conclude that our method well-compares with recent tools at the state-of-the-art.

**Table 4:** Performance of different methods for solvent accessibility prediction on the blind test set described in this study comprising 200 protein sequences.

**Method**	**MCC**	**Q2 %**	**F1 %**
PaleAle 5.0	0.65	82	84
NetSurfP-2.0	0.67	83	81
Our method	0.63	82	82

### Analyzing Distributions of Variated Wild-Type Residues in the Sequence Dataset

**Figure 4:**
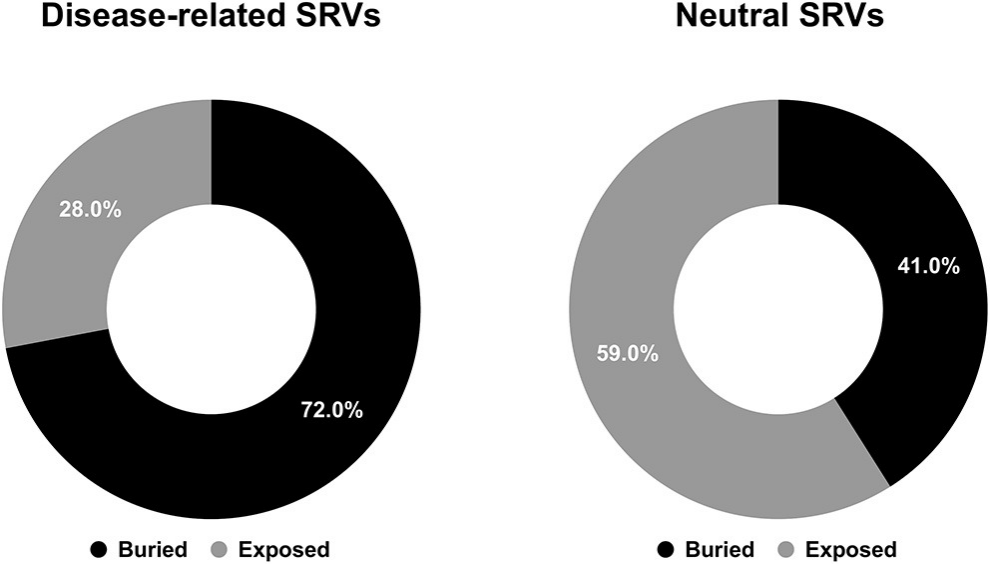
Pie charts showing the fractions of predicted buried/exposed positions disease-related (left) and neutral (right) upon variations in the HVARSEQ dataset, respectively.

After computing solvent accessibility over HVARSEQ, we assessed the proportions of buried and exposed predictions separately on the subsets of residues undergoing disease-related and neutral variations. Results are in Figure 4.

As to the prediction, 72% of disease related SRVs occurs in buried positions and 58% of neutral SRVs affect exposed residues. Interestingly, the proportions of buried/exposed positions for disease and neutral SRVs are in agreement with those assessed on the structural dataset (67% and 64.3%, respectively: compare Figures 1, 4). The result further corroborates the notion that residues undergoing disease-related variations are mainly in buried positions.

**Figure 5:**
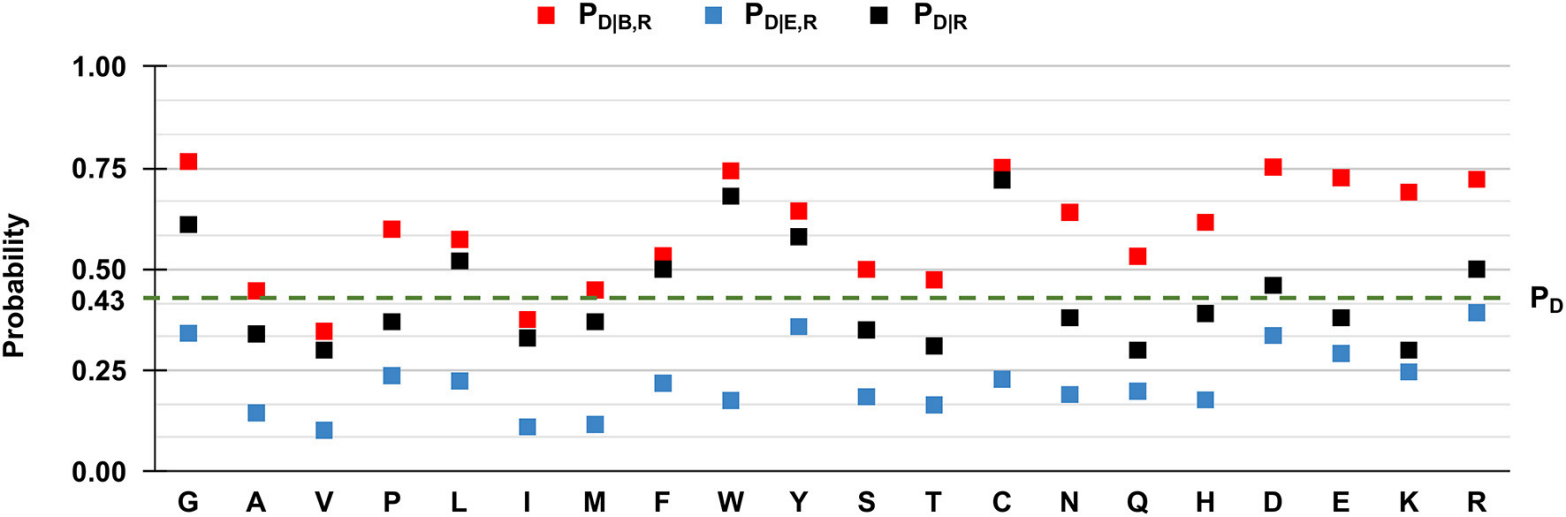
Frequency of disease-related SRVs, depending on the wild type residue and the exposure state in HVARSEQ. Here, buried and exposure states of each residue position have been predicted using the method described in Section Analyzing distributions of variated wild-type residues in the sequence database. **P**_**D**_: the probability of a wild-type residue (position) to be disease associated in the HVARSEQ dataset [see Equation (1)]. **P**_**D|R**_: the conditional probability of being disease related when variated, given a wild-type residue [see Equation (2)]. **P**_**D|B,R**_: the conditional probability of a wild-type residue to be disease related upon variation when buried [see Equation (3)]. **P**_**D|E,R**_: the conditional probability of a wild-type residue R to be disease related upon variation when exposed [see Equation (4)].

We then evaluated P_D|R_, P_D|B,R_, and P_D|E,R_ for all the residue types and results are reported in Figure 5. We also show the baseline probability P_D_ (0.43), which represents the proportion of positions that undergo disease-related variations in the HVARSEQ dataset.

The comparison between P_D|R_ and P_D_, which are both independent from predictions, confirms the finding obtained on the HVAR3D-2.0 dataset: residues such as glycine (G), tryptophan (W), tyrosine (Y), and cysteine (C), when undergoing variation, are more frequently associated to disease than expected from the baseline. In the sequence set, this behavior characterizes also arginine (R) and aspartic acid (D).

Similarly to the structural case, for all residues we have that P_D|B,R_ > P_D|R_ > P_D|E,R_, highlighting that for all residue types, SRVs are more frequently associated to disease when occurring in buried positions than in exposed ones. The tendency is remarkable for the majority of residues, already identified from HVAR3D-2.0 and including asparagine (N), lysine (K), and histidine (H). The analysis on HVARSEQ highlights a difference between P_D|B,R_ and P_D|E,R_ for tryptophan (W) and cysteine (C). However, this discrepancy can be due to prediction errors on these two less abundant (rare) residues in the database. Similarly, to what described for HVAR3D-2.0 (Figure 3), the frequency of disease-related SRVs occurring at valine (V) and isoleucine (I) residues is lower than the baseline, independently of the exposure state.

### Case Study

**Figure 6:**
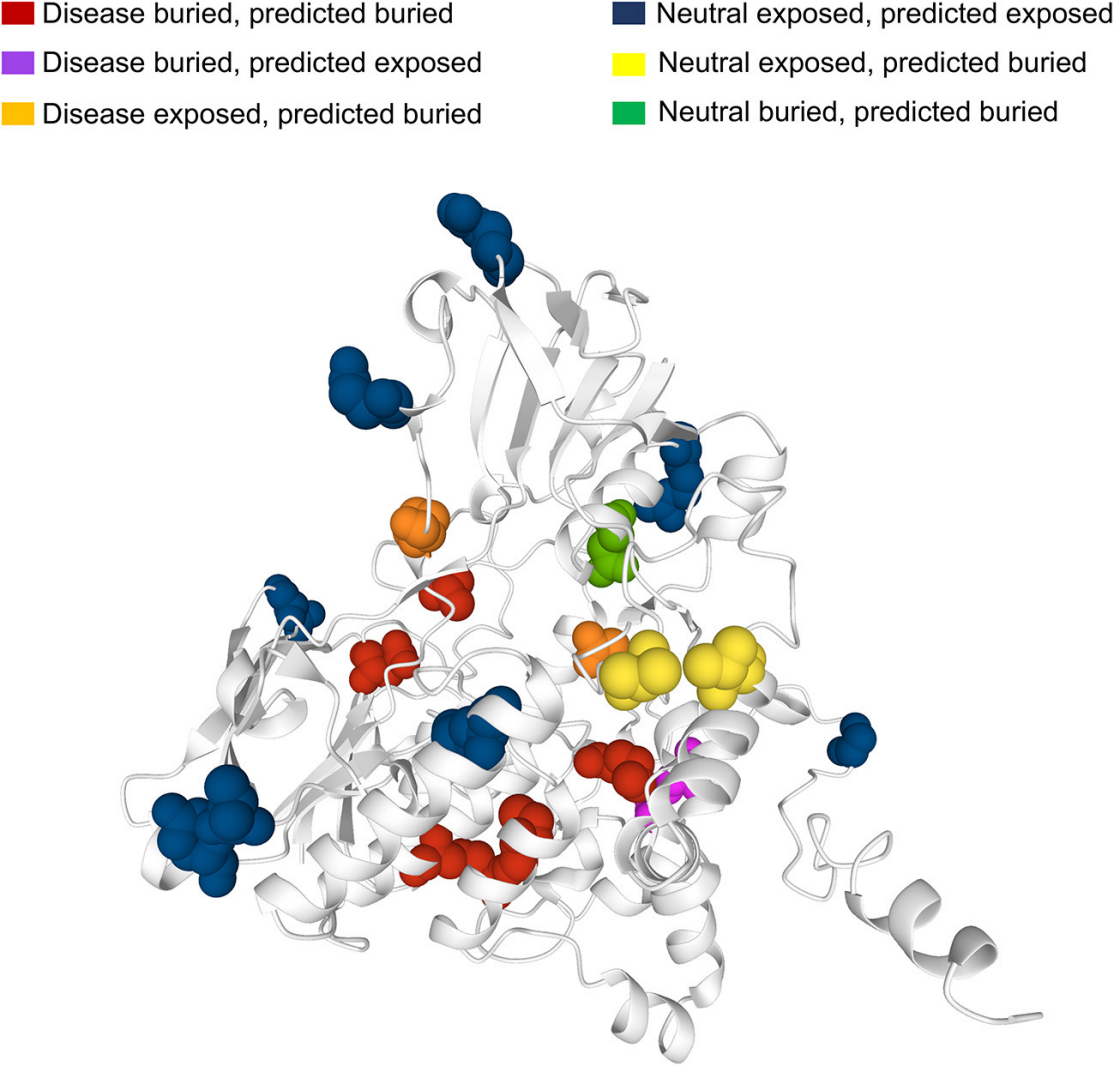
Mapping SASA predictions on a protein model. The model is that of human Dimethylaniline monooxygenase 3 (UniProtKB: P31513) derived from the SWISS-MODEL Repository. Solvent exposure is computed from the available 3D protein model using DSSP. Variation (SVR) positions are highlighted using the spacefill view. In red, buried positions associated to disease-related SRVs and correctly predicted as buried by our method. In magenta, buried disease-related positions wrongly predicted as exposed. In orange, exposed disease-related positions wrongly predicted as buried. In blue, exposed neutral SRV positions correctly predicted as exposed. In yellow, exposed neutral positions wrongly predicted as buried. In green, buried neutral positions correctly predicted as buried.

Many human protein sequences, without any associated three-dimensional (3D) structure, are endowed with models that can be derived from the SWISS-MODEL Repository^3^, directly linked to the protein UniProtKB file. For sake of curiosity, we took advantage of an example to show the 3D location of our sequence-based prediction. In particular, in Figure 6 we show the model of the human Dimethylaniline monooxygenase 3 protein (UniProtKB: P31513)^4^. This protein has 19 SRVs in HVARSEQ, eight of which are disease-related and 11 are neutral. Disease-related SRVs are all associated to Trimethylaminuria (OMIM:602079)^5^, a disease condition resulting from the abnormal presence of large amounts of volatile and malodorous trimethylamine within the body. In Figure 6, we map all the solvent exposure predictions for all SRV positions into the 3D model.

It is evident that the vast majority of disease-related SRVs (6 out of 8) are in buried positions. Of these, five are correctly predicted as buried by our method (in red) while only one is wrongly predicted as exposed (in magenta). Neutral SRVs are mostly exposed (10 out of 11): eight of these are correctly predicted in exposed regions (in blue).

Results illustrate the general trend of what we observed in the structural data set and are consistent with the accuracy of the prediction method.

## Conclusion and Perspective

In this paper, we focus on the solvent accessible surface area, a property of protein residues, firstly described and computed in several biophysical studies, to which Cyrus Chothia contributed (Chothia, 1976). The property, which nowadays can be computed with machine learning based methods, is here exploited in relation to another important problem: the annotation of variations in human proteins as disease related or not. We took advantage of an ample set of human protein structures to observe that indeed disease related variations occur more frequently in buried regions of the proteins than in solvent accessible surfaces. In turn, neutral polymorphisms are characterized by a more frequent solvent exposure. We then proved that with a deep learning method performing at the state of art, the tendency is observable also in the majority of all the wild-type residues undergoing variations that are presently listed in HUMSAVAR. We suggest that the solvent accessible surface area of wild type residues is a distinguished property to be included among those necessary to annotate pathogenic from non-pathogenic variations.

## Data Availability Statement

The original contributions presented in the study are included in the article/Supplementary Material, further inquiries can be directed to the corresponding author/s.

## Author Contributions

RC, PM, and CS: conceptualization and writing. RC, PM, CS, and MM: methodology. MM and CS: software. CS, MM, and PM: data curation and visualization. RC and PM: supervision. All authors contributed to the article and approved the submitted version.

## Conflict of Interest

The authors declare that the research was conducted in the absence of any commercial or financial relationships that could be construed as a potential conflict of interest.

## Footnotes

^1^https://www.ebi.ac.uk/pdbe/docs/sifts/.

^2^ https://www.uniprot.org/docs/humasavar

^3^ https://swissmodel.expasy.org/repository

^4^https://www.uniprot.org/uniprot/P31513.

^5^ https://www.omim.org/entry/60207
